# Case Report: Successful management of acute vincristine overdose in a cat with metastatic gastric lymphoma using therapeutic plasma exchange

**DOI:** 10.3389/fvets.2026.1791728

**Published:** 2026-03-27

**Authors:** Natasha S. Yeh, Rachel Dawn Scott, Michael T. Kato

**Affiliations:** 1 Veterinary Clinical Medicine, College of Veterinary Medicine, University of Illinois Urbana-Champaign, Urbana, IL, United States; 2 VEG ER for Pets, Falls Church, VA, United States; 3 Department of Small Animal Clinical Sciences, Virginia-Maryland College of Veterinary Medicine, Virgina Tech, Blacksburg, VA, United States

**Keywords:** chemotherapy, extracorporeal therapy, overdose, therapeutic plasma exchange, toxicity

## Abstract

A 10-year-old male castrated Siamese cat with gastric large B-cell lymphoma was presented following an accidental overdose of vincristine, four times the intended dose. The initial management involved administering intravenous lipid emulsion. Extracorporeal therapy was implemented to reduce plasma vincristine concentrations, beginning with therapeutic plasma exchange (TPE) and followed by hemoperfusion (HP). Vincristine clearance was not confirmed through pre- and post-treatment serum levels due to a laboratory handling error. The patient became apneic at the initiation of HP, prompting discontinuation of treatment and completion of TPE alone. Post-TPE management combined intensive supportive care with the administration of cholestyramine, calcium folinate, and granulocyte colony-stimulating factor. The cat survived to discharge, suggesting a potential role for extracorporeal therapy in managing chemotherapeutic toxicity. However, the absence of pre- and post-treatment serum vincristine levels prevents a definitive assessment of therapeutic efficacy.

## Introduction

Chemotherapy errors, by either overdose or incorrect medication, can pose a potentially serious risk of harm to patients. Chemotherapy overdose is defined as the administration of a chemotherapeutic medication at a dose that exceeds the intended dose by 10% or is given at a shorter interval than planned, and these occurrences are rare in veterinary medicine (1). A 2021 retrospective study identified 28 cases of chemotherapy overdoses in dogs and 3 cases in cats reported through the American College of Veterinary Internal Medicine’s oncology and internal medicine listserv (1).

Vincristine, a vinca alkaloid, is a commonly used chemotherapeutic agent that causes mitotic arrest in rapidly dividing cells by binding microtubule proteins (2). Overdoses of vincristine have been more frequently described in felines, leading to significant morbidity and mortality, with limited treatment options beyond supportive care (1, 3). A 6-year-old Burmese cat that received a 10-fold vincristine overdose was treated with calcium folinate but died after 72 h. A 2021 retrospective study reported that two of three cats that received a 2.4-fold vincristine overdose were euthanized due to clinical decline within 36 h (1, 3). An 11-year-old Manx cat that received a 4-fold vinblastine overdose survived following treatment with recombinant human granulocyte-colony stimulating factor (4).

Extracorporeal therapy (ECT) is a blood purification technique utilized to remove toxic substances from the body through hemodialysis, hemoperfusion, or therapeutic plasma exchange (TPE) (5). Vincristine has a large volume of distribution and moderate protein binding, which limits the effectiveness of ECT. However, ECT has been used in the treatment of chemotherapy overdoses in both canines and humans (6–10). To the authors’ knowledge, this is the first reported case of utilizing attempted and partially completed extracorporeal therapy for chemotherapeutic overdose in a cat. This case demonstrates the use of partial extracorporeal therapy with TPE alone in the management of vincristine overdose, resulting in short-term survival until discharge. However, due to incomplete HP and insufficient drug concentration data, the clearance of vincristine and the therapeutic efficacy cannot be determined.

## Case description

An approximately 10-year-old male castrated Siamese with a 4-month history of hyporexia and vomiting was diagnosed with gastric large B-cell lymphoma. An abdominal ultrasound revealed a focal 8.1 mm gastric mural thickening along the lesser curvature with regional lymphadenopathy and mild bilateral degenerative renal changes. Initial treatments included prednisolone (0.75 mg/kg PO q24 h), and a follow-up ultrasound 14 days later showed the focal gastric thickening had decreased to 5.2 mm. One month later, a repeat ultrasound revealed that the gastric mural thickening had increased to 15 mm x 5.3 mm. As a result, surgical gastric resection of the focal thickening was recommended. Two small, adjacent mass lesions were identified near the cardia and removed en bloc, along with a gastric lymph node. Histopathological examination of the gastric tissues revealed regionally extensive mural infiltrative malignant round-cell sarcoma, with a mitotic count of 18 and incomplete resection margins. Lymph node histopathology revealed severe, generalized large cell lymphoid hyperplasia, raising suspicion for high-grade malignant lymphoma. Immunohistochemical staining identified the neoplasm as a large B-cell lymphoma. One month post-operatively, an ultrasound showed a 1.7-cm mass-like thickening of the gastric cardia wall. Treatment was initiated with L-asparaginase, followed by the CHOP protocol of vincristine, cyclophosphamide, doxorubicin, and prednisolone. Prednisolone was increased to 2 mg/kg PO q24 h on a tapering course. During the administration of the second vincristine dose, the patient inadvertently received another dose of vincristine (2.65 mg/m^2^) intravenously, which was approximately four times the intended dose; for the chronological purposes of this case report, the day of vincristine overdose is considered day 0. Serial blood tests conducted during the cat’s CHOP protocol is documented in Table 1. On day 0, a complete blood count (CBC) performed at the referring hospital revealed a normal white blood cell count (WBC; 15.06 K/μL, reference interval (RI): 2.87–17.02 K/μL), a normal platelet count (297 K/μL, RI: 151–600 K/μL), and mild anemia (hematocrit 29.6%, RI: 30.3–52.3%). The patient presented to the emergency services at a university teaching hospital for extracorporeal therapy, supportive care, and hospitalization.

**Table 1:** Complete blood count values from the referring hospital from the start of chemotherapy to the day of vincristine overdose (day 0).

Timeline	Day of L-asparaginase	Day of first vincristine	1 week post first vincristine	Day of cyclophosphamide	Day of vincristine overdose
Day	−26	−21	−14	−7	0
White blood cell count (2.87–17.02 K/μl)	11.12	15.02	14.5	14.73	15.06
Neutrophil count (2.30–10.29 K/μl)	9.06	13.09	11	12.75	12.86
Hematocrit (30.3–52.3%)	28.8	29.9	27.1	28.5	29.6
Reticulocytes (3.0–50.0 K/μl)	23.9	41.9	118.8	29.2	126.3
Platelet count (151–600 K/μl)	231	445	89 (with significant clumping)	433	297

On presentation, the patient was bright and alert with a rectal temperature of 102.8 °F (39.3 °C), a heart rate of 180 beats/min, a respiratory rate of 40 breaths/min, pale pink mucous membranes, and a capillary refill time of 1–2 s. A grade II/VI left parasternal systolic heart murmur was auscultated. Initial point-of-care blood work included packed cell volume (PCV) of 32%, total solids (TS) of 7.7 mg/L, blood glucose (BG) of 186 mg/dL, and an elevated lactate of 3.5 mmol/L. Serum biochemistry showed mild ALP elevation (74 IU/L; RI: 11–51 IU/L), total hypocalcemia (8.8 mg/dL; RI: 9.2–10.7 mg/dL), hypophosphatemia (2.7 mg/dL; RI: 2.9–6.3 mg/dL), and hypoalbuminemia (3.0 g/dL; RI: 3.1–4.5 g/dL). Blood type was A. A poison control case was opened through a commercially available animal poison control center (ASPCA Animal Poison Control Center, personal communication, April 2022). Abdominal ultrasound revealed a gastric body and fundic mass, a mildly enlarged (0.19 cm) pyloroduodenal lymph node, and a hypoechoic pancreas with associated hyperechoic mesentery. The patient was hospitalized for intralipid emulsion (1.5 mL/kg over 10 min IV followed by constant rate infusion (CRI) 15 mL/kg over 1 h) prior to extracorporeal treatments.

A 7 Fr, 16 cm double-lumen hemodialysis catheter (MILA, Kentucky, United States) was placed under sedation in the right jugular vein, with positioning confirmed in the right atrium via thoracic radiographs. TPE was performed using a centrifugal platform (Spectra Optia Apheresis System, Terumo, Lakewood, Colorado, USA) with a prescription of two plasma volumes exchanged using fresh-frozen plasma at a volume of 348 mL. The plasma volume to be exchanged was calculated using the formula: Plasma volume(mLs)=Total Body Volume(TBV)x(1−Hematocrit) . The TBV was assumed to be 60 mL/kg, and the hematocrit used was 32%. The circuit was blood-primed with 111 mL of packed red blood cells and 39 mL of fresh-frozen plasma. Sodium citrate was used for circuit anticoagulation, and dexmedetomidine (1 mcg/kg/h IV CRI) was administered to the patient for procedural sedation. The patient’s body weight was initially entered incorrectly as 5.26 kg instead of 4.26 kg, and this was corrected in the last half hour of treatment. A total fluid balance error alarm indicated greater plasma loss than return. At the time the body weight discrepancy was identified, fresh-frozen plasma was used exclusively in the exchange to provide oncotic support, given the lack of availability of frozen plasma or albumin products. Despite this error, a two-time plasma volume exchange was achieved and an additional 6 mL of sodium citrate was administered, with no hemodynamic or biochemical consequences observed at the time. Circuit volume calculations and blood priming volumes were based on the correct body weight. The total fresh-frozen plasma replacement volume was 348 mL and plasma removed was 420 mL with a treatment time of 3 h and 40 min and a partial rinse back of 44 mL. The error resulted in an over-exchange with a net fluid loss of 72 mL relative to the intended prescription.

Hemoperfusion was then initiated using an intermittent hemodialysis platform (Pheonix X36 Hemodialysis System, Baxter, Deerfield, IL, United States) with a 50 mL hemoperfusion cartridge (VetResQ® device, Cytsorb, New Jersey, United States). Systemic anticoagulation was started using a continuous heparin infusion. Within 15 min of starting hemoperfusion, the patient acutely became apneic. Electrocardiogram at the time showed a normal sinus rhythm at 160 beats per minute. Cardiopulmonary resuscitation was initiated with chest compressions and endotracheal intubation using a size 4.5 endotracheal tube. Hemoperfusion was terminated, and a rinse back was performed. Chest compressions were performed for approximately 2 min. The patient began spontaneously breathing within 4 min and was then extubated. The patient was mentally obtunded to stuporous and showed improvement to a quiet, appropriate mentation over the course of 4 h.

The patient was hospitalized with medical therapies including 0.45% NaCl (30 mL/kg/day IV), fresh-frozen plasma (20 mL/kg/day), maropitant (1 mg/kg IV q24 h), metoclopramide (2 mg/kg/day IV CRI), prednisolone (1.1 mg/kg PO q24 h), gabapentin (10 mg/kg PO q8-12 h as needed), cholestyramine (4 g PO q12 h), and leucovorin (folinic acid; 2 mg/kg IV q3 h for first 12 h and then q6 h for 48 h).

Serial complete blood counts were performed (Table 2), which showed an initial segmented neutrophil count of 22,000 K/μL (RI: 2.773–6.975 K/μL). On day 2, filgrastim (5 mcg/kg SQ q24 h) was initiated. At this time, the CBC showed a segmented neutrophil count of 17.766 K/μL. On day 3, the patient was febrile, and the CBC showed acute changes including severe leukopenia (WBC 1.54 K/μL; RI: 3.77–16.73 K/μL), neutropenia (1.217 K/μL), lymphopenia (0.077 K/μL; RI: 0.415–4.996 K/μL), monocytopenia (0.046 K/μL; RI: 0.068–0.780 K/μL), thrombocytopenia (44 K/μL; RI: 198–434 K/μL), and progressive non-regenerative anemia (hematocrit 18.7%; RI: 33.0–51.0%). Unasyn (22 mg/kg IV q8 h) and ciprofloxacin (10 mg/kg IV q24 h) were initiated. A nasogastric tube was placed for nutritional support. On day 4, the patient’s neutrophil count dropped to 0.140/ K/μL with a WBC count of 0.39 K/μL. The patient had persistent hyperglycemia (BG > 300 mg/dL), with blood ketones measured at 2.6 mmol/L. As a result, a regular insulin infusion (0.05 units/kg/h) was initiated. On day 11, the patient underwent an esophagostomy tube procedure, a hydrolyzed protein-blended diet was formulated, and prednisolone was discontinued. On day 13, the day prior to discharge, the CBC showed improvement, with an elevated neutrophil count of 26.401 K/μL and a normal platelet count of 286 K/μL (Table 2).

**Table 2:** Serial complete blood count values of the cat during hospitalization from days 1 to 13, with discharge on day 14.

Day	+1	+2	+3	+4	+5	+6	+7	+8	+9	+13
White blood cell count (3.77–16.73 K/μl)	23.48	18.9	1.54	0.39	2.35	18.09	11.6	20.69	33.14	27.79
Neutrophil count (2.773–6.975 K/μl)	22.07	17.76	1.21	0.14	1.481	15.738	8.352	18.414	29.495	26.4
Lymphocyte count (0.415–4.996 K/μl)	0.235	0.378	0.077	0.055	0.141	-	0.464	0.621	1.657	0.834
Monocyte count (0.068–0.780 K/μl)	0.47	-	0.046	0.047	0.024	0.543	0.928	0.621	0.663	0.834
Hematocrit (33.0–51.0%)	27.8	22.2	18.7	23.3	17.1	22	18.4	15.3	18.5	19
Reticulocytes (12–64 K/μl)	84	13	15	21	16	24	20	12	24	39
Platelet count (198–434 K/μl)	592 (with excessive clumping)	49	44	75	34	40	35	30	68 (with rare clumping)	286

Throughout the 15 days of hospitalization, the patient became progressively anemic, requiring a total of two crossmatch-compatible whole blood transfusions and one crossmatch-compatible packed red blood cell transfusion. On day 14, PCV was 21%, and TS was 6.5 mg/L. The patient was discharged with a prescription of maropitant, ondansetron, metoclopramide, mirtazapine, and Visbiome.

## Follow-up and outcomes

The patient presented to the oncology service for a follow-up 17-day post-vincristine overdose. An abdominal ultrasound showed altered gastric wall layering, prioritizing secondary surgical changes and no overt gastric mas. Recheck PCV was 26% and TS was 7 g/dL. On day 23, the patient was restarted on prednisolone at a dosage of 1 mg/kg/day due to anorexia and for the treatment of B-cell lymphoma. Fructosamine levels were within the normal reference range, following previous hyperglycemia during hospitalization. On day 44, an abdominal ultrasound showed thickening of the gastric fundus suspicious for neoplastic growth and hypoechoic liver nodules. On day 46, the patient presented with acute ataxia, a left head tilt, persistent horizontal nystagmus, and positional rotary nystagmus, with intact conscious proprioception and bright, appropriate mentation. The owner noted intermittent dragging of his left hind limb. No murmurs or arrhythmias were detected during auscultation, and the quality of his left femoral pulse was fair. All vital signs were within normal limits. The Doppler pressure in the left and right hind limbs was 95–105 mmHg. The paired BG and lactate levels in the left and right hind limbs were similar to those in the left forelimb. The patient showed signs of peripheral vestibular dysfunction and was discharged with a prescription for gabapentin (7 mg/kg PO q12 h). The owners re-presented with the patient 24 h later and opted for humane euthanasia on day 47 due to the acute onset of neurological signs, the patient’s multiple comorbidities, and poor long-term prognosis.

**Figure 1:**
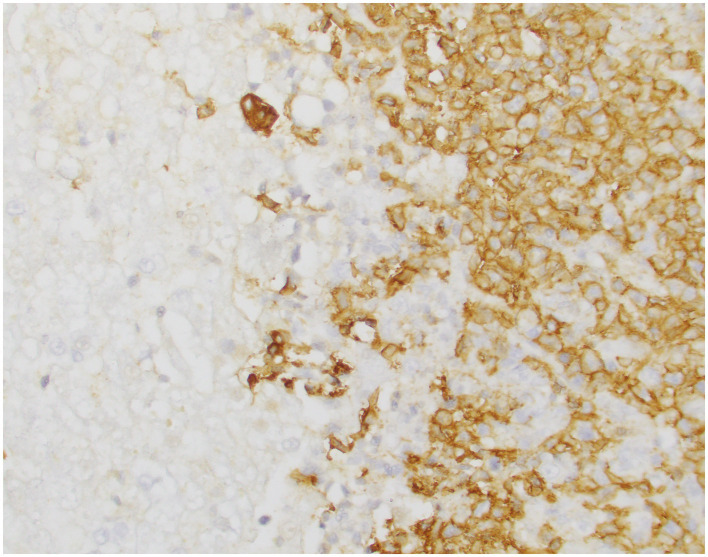
CD20 immunohistochemical staining of liver tissue, highlighting B-cell lymphoma infiltrates. Magnification: 40X.

An autopsy identified a 2-cm ulcerated plaque expanding into the gastric mucosa and multifocal 1–2 mm pale, raised nodules throughout all hepatic lobes. The tympanic bullae were unremarkable. Histopathological examination showed that the gastric mucosa and submucosa were expanded with sheets of atypical large lymphocytes with similar collections of atypical lymphocytes in the liver nodules, colon, renal cortex, heart, meninges of the forebrain at the level of the hippocampus, the midbrain at the level of the choroid plexus, surrounding the cerebellum, and along the lateral brainstem, suggestive of widely metastatic disease. Immunohistochemical staining for CD20 was positive in liver and gastric tissues, indicating the presence of B-cell lymphoma (Figure 1).

## Discussion

Therapeutic plasma exchange for vincristine toxicity in a cat has not been reported in a clinical setting. Moreover, this case supports the literature on the use of cholestyramine, calcium folinate, and granulocyte colony-stimulating factor in cases of vinca alkaloid overdose in cats and dogs (1, 3, 4, 11). Due to the limited number of reported cases of vincristine overdose that document survival to hospital discharge, the primary clinical outcome for this patient was defined as survival to discharge. Vincristine-induced cytopenias developed, including a 3-day neutropenia and an 8-day thrombocytopenia. Both were resolved prior to discharge. Anemia was present at admission, decreased to a nadir hematocrit of approximately 50% of the initial value, and required three transfusions. No clinical or postmortem evidence of vincristine-associated neurotoxicity, hepatotoxicity, renal tubular necrosis, or pulmonary edema was identified. Transient obtundation on the first day of admission was attributed to a 4-min respiratory arrest. The neurological signs observed on day 46 were attributed to neoplastic infiltration of the central nervous system, as evidenced by necropsy findings, rather than to delayed vincristine neurotoxicity. Postmortem evaluation of the bone marrow did not demonstrate depletion consistent with vincristine toxicity. Folinic acid is hypothesized to counteract the methotrexate’s inhibition of dihydrofolate reductase and thymidylate synthetase in DNA synthesis, although its efficacy against vincristine toxicity is debated (3, 11–13). Neutropenia nadir varies by individual and chemotherapeutic agent. With cyclophosphamide and vincristine, neutropenia nadir typically occurs between 5 and 10 days after administration (14). The patient’s white blood cell nadir occurred approximately day 4. The administration of cyclophosphamide 11 days before the observed nadir complicated the interpretation, as this nadir may represent late-onset bone marrow suppression from cyclophosphamide, early effects of vincristine, or a combination of the two (15, 16). A cyclophosphamide overdose following vincristine administration in a dog with multicentric lymphoma did not result in synergistic toxicity, where the combined toxic effects would exceed those of each drug alone (14). It is reasonable to speculate that a similar principle may apply to the patient in this case report, who received a therapeutic dose of cyclophosphamide 7 days before the vincristine overdose. However, attributing neutropenia to a single agent is not possible due to the timeline of chemotherapeutic drug administration. The cat lived approximately 47 days post-vincristine overdose. The autopsy showed that the systemic decline was due to progressive disseminated lymphoma and was not directly related to the vincristine overdose.

Vincristine overdose in humans has been documented to cause bone marrow suppression, gastrointestinal disorders, and neurotoxicity (12, 17, 18). A report describing three pediatric lymphoblastic leukemia patients who received an accidental overdose of vincristine and subsequently underwent a double-volume exchange, along with prophylactic phenobarbital and folinic acid, reported peripheral neuropathy beginning on day 4, bone marrow toxicity on day 5, gastrointestinal toxicity on days 6 and 7, and hypertension on days 7 and 8 (16). Another case report of a 10-fold vincristine overdose in an 18-year-old patient described successful plasmapheresis treatment (19). These reports support the effectiveness of extracorporeal therapy as a treatment for vincristine overdose.

Extracorporeal therapy modalities are becoming more available in veterinary medicine, and their use for acute toxin removal is well-known (5). The use of extracorporeal therapy for chemotherapy overdose is currently limited (1, 6). Vincristine has an approximate volume of distribution of 1.9 L/kg and is 75% protein-bound. It has a molecular weight of 824.96 Daltons (Da) and a plasma half-life of 11 h (20, 21). In humans, vincristine clearance has been shown to be dose-dependent, and decreased clearance is associated with elevated alkaline phosphatase levels (22). A study comparing vincristine pharmacokinetics observed similar characteristics in the short first half-life and rapid tissue accumulation between dogs, monkeys, and humans (23). There is a lack of literature regarding vincristine pharmacokinetic data in cats. TPE involves the separation and replacement of a patient’s blood plasma to remove circulating substances that have high protein binding (>80%), a high molecular weight (>40,000 Da), and a low volume of distribution (<0.5 L/kg) (5, 24, 25). Hemoperfusion is the passage of a patient’s blood through an adsorbent column to non-selectively remove solutes that are highly protein-bound (>80%), of low-to-high molecular weight (<40,000 Da), and has low-to-moderate volume of distribution (>0.5 L/kg) (5, 26, 27). The initial treatment strategy for this patient involved using TPE as a salvage procedure to remove as much vincristine as possible from the plasma at the time of treatment, followed by HP treatment over 12 to 24 h. Due to vincristine’s large volume of distribution, TPE alone may not provide adequate clearance; therefore, continuous HP therapy was attempted. The patient became hemodynamically unstable at the start of HP treatment and became apneic. Hemoperfusion was discontinued even after the patient began spontaneously breathing. Although the TPE treatment was interrupted due to the fluid balance alarm, the therapeutic objective of two plasma exchanges was achieved. The patient’s apnea may have resulted from hypovolemic shock caused by incorrect TPE settings, resulting in plasma removal exceeding the replacement and removal of whole blood from the hemoperfusion circuit and filter. Other potential causes include reactions to the contents of the filter or anticoagulation-related hemorrhage; however, neither of these was evident in this case. Extracorporeal therapy carries an inherent risk of hypovolemia, particularly in smaller-sized patients. This risk can be mitigated through careful monitoring, pre-treatment blood work evaluation, circuit volume calculations, and strategies such as blood priming. Serum vincristine levels were collected pre- and post-TPE treatment, but shipping and receiving errors prevented the samples from being analyzed. The patient was hospitalized for 15 days post-TPE treatment and ultimately survived to discharge.

## Conclusion

To the authors’ knowledge, this is the first report documenting the use of extracorporeal therapy to treat vincristine overdose in a cat. Although the intended combined TPE and HP therapy was not achieved, this patient survived to discharge with a singular TPE treatment, along with aggressive supportive measures and specific therapies that included cholestyramine, calcium folinate, and granulocyte colony-stimulating factor. The cat survived to day 47 following the vincristine overdose. A key limitation of this case report is that the effectiveness of TPE in treating vincristine overdose remains uncertain without drug concentration data. Chemotherapy overdose cases lack representation in the veterinary literature, and both the medical management and the feasibility of extracorporeal therapy are reported here to aid in the clinical approach for future cases of chemotherapy overdoses.

## Data Availability

The original contributions presented in the study are included in the article/supplementary material, further inquiries can be directed to the corresponding author.

## References

[1] Musser ML Curran KM Flesner BK Johannes CM. A retrospective evaluation of chemotherapy overdoses in dogs and cats. Front Vet Sci. (2021) 8:8. doi: 10.3389/fvets.2021.718967, 34631850 PMC8492923

[2] Hahn KA. Vincristine sulfate as single-agent chemotherapy in a dog and a cat with malignant neoplasms. J Am Vet Med Assoc. (1990) 197:504–6. 2211297

[3] Hughes K Scase TJ Ward C Polton GA. Vincristine overdose in a cat: clinical management, use of calcium folinate, and pathological lesions. J Feline Med Surg. (2009) 11:322–5. doi: 10.1016/j.jfms.2008.06.006, 18774324 PMC10911459

[4] Grant IA Karnik K Jandrey KE. Toxicities and salvage therapy following overdose of vinblastine in a cat. J Small Anim Pract. (2010) 51:127–31. doi: 10.1111/j.1748-5827.2009.00864.x 20137000

[5] Foster JD. Extracorporeal therapies in the emergency room and intensive care unit. Vet Clin North Am Small Anim Pract. (2020) 50:1215–36. doi: 10.1016/j.cvsm.2020.07.014 32981594

[6] Groover J Londoño LA Tapia-Ruano K Iacovetta C. Extracorporeal blood purification in acutely intoxicated veterinary patients: a multicenter retrospective study (2011–2018): 54 cases. J Vet Emerg Crit Care. (2022) 32:34–41. doi: 10.1111/vec.13100, 34897946

[7] Pardo M Lanaux T Davy R Bandt C. Use of charcoal hemoperfusion and hemodialysis in the treatment of methotrexate toxicosis in a dog. J Vet Emerg Crit Care. (2018) 28:269–73. doi: 10.1111/vec.12719, 29727524

[8] Relling MV Stapleton FB Ochs J Jones DP Meyer W Wainer IW . Removal of methotrexate, leucovorin, and their metabolites by combined hemodialysis and hemoperfusion. Cancer. (1988) 62:884–8. 3261621 10.1002/1097-0142(19880901)62:5<884::aid-cncr2820620506>3.0.co;2-a

[9] Spiller M Marson P Perilongo G Farina M Carli M Bisogno G. A case of vinblastine overdose managed with plasma exchange. Pediatr Blood Cancer. (2005) 45:344–6. doi: 10.1002/pbc.20284, 15602712

[10] Yamada Y Ikuta Y Nosaka K Miyanari N Hayashi N Mitsuya H . Successful treatment of cisplatin overdose with plasma exchange. Case Rep Med. (2010) 2010:802312. doi: 10.1155/2010/802312, 20300587 PMC2837905

[11] Poirier M Blong AE Walton RAL. Successful management and recovery of a dog with immune-mediated thrombocytopenia following vincristine overdose. J Vet Emerg Crit Care. (2022) 32:539–44. doi: 10.1111/vec.13187, 35129277 PMC9546371

[12] Thomas L Braat P Somers R Goudsmit R. Massive vincristine overdose: failure of leucovorin to reduce toxicity. Cancer Treat Rep. (1982) 66:1967–9. 6982752

[13] Beer M Cavalli F. Vincristine overdose: treatment with and without leucovorin rescue. Cancer Treat Rep. (1983) 67:611–752. 6603267

[14] Ferrer-Jorda E Rodriguez-Piza I. Complete recovery of a cyclophosphamide overdose after vincristine administration in a dog. Vet Rec Case Rep. (2023) 11:e591. doi: 10.1002/vrc2.591

[15] Cunha SC dos S Silva FB Corgozinho KB Silva KVGC da Ferreira AMR Retrospective study of adverse events of chemotherapy in cats Acta Sci Vet (2018) 46 12–12 doi: 10.22456/1679-9216.81801

[16] Kosmidis HV Bouhoutsou DO Varvoutsi MC Papadatos J Stefanidis CG Vlachos P . Vincristine overdose: experience with 3 patients. Pediatr Hematol Oncol. (1991) 8:171–8. 1863543 10.3109/08880019109033445

[17] Schulmeister L. Preventing vincristine sulfate medication errors. Oncol Nurs Forum. (2004) 31:E90–8. doi: 10.1188/04.ONF.E90-E98, 15378106

[18] Chae L Moon HS Kim SC. Overdose of vincristine: experience with a patient. J Korean Med Sci. (1998) 13:334–8. 9681818 10.3346/jkms.1998.13.3.334 PMC3054504

[19] Pierga JY Beuzeboc P Dorval T Palangie T Pouillart P. Favourable outcome after plasmapheresis for vincristine overdose. Lancet. (1992) 340:185. 10.1016/0140-6736(92)93272-o 1352603

[20] Phalen DN Frimberger A Pyecroft S Peck S Harmsen C Lola S . Vincristine chemotherapy trials and pharmacokinetics in Tasmanian devils with Tasmanian devil facial tumor disease. PLoS One. (2013) 8:e65133. doi: 10.1371/journal.pone.0065133, 23762298 PMC3675106

[21] Cancer BC. Drug Index. (2025). Available online at: https://www.bccancer.bc.ca:443/health-professionals/clinical-resources/cancer-drug-manual/drug-index (Accessed November 23, 2025).

[22] Van den Berg HW Desai ZR Wilson R Kennedy G Bridges JM Shanks RG. The pharmacokinetics of vincristine in man: reduced drug clearance associated with raised serum alkaline phosphatase and dose-limited elimination. Cancer Chemother Pharmacol. (1982) 8:215–9. 7105384 10.1007/BF00255487

[23] El Dareer SM White VM Chen FP Mellet LB Hill DL. Distribution and metabolism of vincristine in mice, rats, dogs, and monkeys. Cancer Treat Rep. (1977) 61:1269–77. 412588

[24] Samtleben W Mistry-Burchanardi B Lennertz A Bosch T. Therapeutic plasma exchange in the intensive care setting. Ther Apher Dial. (2001) 5:351–257. doi: 10.1046/j.1526-0968.2001.00383.x 11778919

[25] Kaplan AA. Therapeutic plasma exchange: a technical and operational review. J Clin Apher. (2013) 28:3–10. doi: 10.1002/jca.21257, 23420589

[26] Ronco C Bellomo R. Hemoperfusion: technical aspects and state of the art. Crit Care. (2022) 26:135. doi: 10.1186/s13054-022-04009-w, 35549999 PMC9097563

[27] Kaplan AA. Moderator’s view: high-volume plasma exchange: pro, con and consensus. Nephrol Dial Transplant. (2017) 32:1464–7. doi: 10.1093/ndt/gfx091, 29059395

